# An empirical evaluation of the common disease-common variant hypothesis

**DOI:** 10.1186/1753-6561-1-s1-s5

**Published:** 2007-12-18

**Authors:** Gary K Chen, Eric Jorgenson, John S Witte

**Affiliations:** 1 Department of Epidemiology and Biostatistics, Institute for Human Genetics, Universityof California, San Francisco, California 94143-0794, USA

## Abstract

While genome-wide linkage studies have been successful in mapping variants underlying rare monogenic disorders, genome-wide association studies may be more appropriate for detecting common variants of modest effects that underlie common disorders. To this end, we were interested in determining whether genetic variants associated with a phenotype differed depending on whether they were within or outside of regions linked to the phenotype. In particular, we compared allele frequencies and effect sizes between associated single-nucleotide polymorphisms within and outside of linkage regions using the Genetic Analysis Workshop 15 Problem 1. We did not find any statistically significant differences between these two sets. However, our power calculations show that these results may be inconclusive.

## Background

Linkage analyses have proven effective for the identification of many traits that display a simple Mendelian inheritance pattern. However, these studies have been less successful in deciphering complex traits, potentially due to differing distributions of allele frequencies and effect sizes underlying these diseases across different populations. The common-disease common-variant (CDCV) hypothesis posits that common traits are most likely due to common variants with small to modest effects on disease that may have escaped stringent selection pressure. Hence, linkage analysis can provide greater power to capture a rare variant with a large effect, whereas association studies may have greater power to detect common variants with small effects [1].

It remains unclear, however, whether associated variants will differ depending on whether they are within or outside of regions exhibiting linkage to the phenotype of interest. To investigate this, we compared the distributions of allele frequencies and effect sizes between associated and linked single-nucleotide polymorphisms (SNPs) using data from the Genetic Analysis Workshop 15 (GAW15) Problem 1 and publicly available information from the HapMap project. In particular, we conducted genome-wide linkage and association analyses on a large number of quantitative gene expression phenotypes. Then we evaluated the allele frequencies and estimated effect sizes of SNPs that are linked and/or associated with expression levels.

## Methods

For genome-wide linkage analyses, we obtained data from the GAW 15 Problem 1 data set, which included 3554 log_2_-transformed expression values and 2882 SNP genotypes for 14 Centre d'Etude du Polymorphisme Humain Utah (CEPH) families consisting of 194 individuals [2]. We then undertook variance-components-based multipoint linkage analyses on all autosomes for each of the continuous traits using SOLAR [3].

For the genome-wide association analyses, we obtained genotype data based on Build 35 of the human genome from the HapMap Project website [4]. 3,719,872 SNP genotypes from thirty trios (families with two parents and a child) from the CEPH family data repository were available for this build on the HapMap website. In order to reduce the number of SNPs that were correlated through linkage disequilibrium, we retained only genotypes of markers included in the Illumina 550 k marker panel [5]. Power was calculated for the association analysis using the software Quanto [6].

The same 3554 log_2_-transformed expression phenotypes used for linkage analyses were also used for our association analyses. These were obtained for 57 unrelated founders of the 14 CEPH families from the NCBI Gene Expression Omnibus website [7] using accession number GSE2552. We assumed the simplest genetic model, where any given allele was equally penetrant (no dominance effect). Thus, an additive penetrance model was specified and heterozygote genotypes coded as the midpoint between the values of the two homozygote genotypes. SNPs that were not polymorphic across the 57 phenotyped individuals were omitted from association analyses. A linear model was fit for all possible SNP-phenotype pairs using the genotype and expression data. The resulting regression coefficient was tested for association using a Wald statistic.

## Results

Linkage was evaluated across a 1-cM interval grid for 3550 phenotypes (SOLAR failed to converge for four phenotypes). In light of the large number of phenotypes evaluated, we adjusted a standard LOD > 3.3 cutpoint for "significance" [8] for the number of phenotypes, giving a threshold of 4.99. Six chromosomal regions (1p13-q23, 7q36, and 9q33-34, 14q24-32, 16p13, and 18p11) were linked under the adjusted threshold, where a region was deemed "linked" if a contiguous sequence of grid positions had a linkage score that exceeded the threshold for at least one phenotype. To allow for comparison with the association results, SNPs from the Illumina panel falling within these regions were then assumed to be linked. We observed a total of 15,976 "linked" SNPs. The average number of SNPs for each linkage region was 2663, with a median of 2719.

Association was tested between 526,935 SNPs and the 3550 phenotypes. We initially specified a two-tailed significance threshold of Z = 6.66 (*p *< 1.3 × 10^-11^), which corresponds to a Bonferroni correction across the large number of comparisons. Of course, this correction is likely to be conservative because some of the SNPs – as well as the phenotypes – are correlated. A SNP was deemed "associated" if it surpassed the threshold for any phenotype. Here we detected a total of 227 associated SNPs.

To explore our hypothesis that the distribution characteristics of associated SNPs might differ depending on whether they were within or outside of linkage regions, we classified the full set of SNPs into four groups: linked and associated, only associated, only linked, and neither linked nor associated (Table 1). The allele frequencies did not differ among the first two groups. Looking at effect sizes – as measured by regression coefficients for association (i.e., β) – we observed a slightly higher effect size for SNPs both associated and in linkage regions versus those only associated (first two rows of Table 1). This difference was not, however, statistically significant.

**Table 1:** Comparison of SNPs linked versus those associated with 3550 phenotypes

Thresholds			SNP category^a^			Minor allele frequencies			β Coefficients (for associated SNPs)		
				
Phenotypes	Linkage (LOD)	Assoc (Z)	A	L	Number	Range	Median	Mean	Range	Median	Mean
3550	4.99	6.66	+	+	6	0.01–0.02	0.01	0.01	2.4–5.7	4.7	4.4
			+	-	221	0.01–0.5	0.01	0.03	0.36–5.5	3.8	3.5
			-	+	15970	0.01–0.5	0.24	0.25			
			-	-	510738	0.01–0.5	0.23	0.25			
											
355^b^	4.03	6.32	+	+	12	0.01–0.38	0.02	0.05	0.4–5.7	3.5	3.3
			+	-	293	0.01–0.5	0.01	0.04	0.3–5.4	3.2	3.1
			-	+	19276	0.01–0.5	0.23	0.25			
			-	-	507354	0.01–0.5	0.23	0.25			
											
108^c^	3.54	6.13	+	+	21	0.01–0.38	0.02	0.06	0.27–5.7	2.4	2.7
			+	-	335	0.01–0.5	0.01	0.04	0.16–5.4	3.2	3.1
			-	+	29387	0.01–0.5	0.23	0.25			
			-	-	497192	0.01–0.5	0.23	0.25			
											
3550	3.3^d^	5.34^d^	+	+	82	0.01–0.5	0.07	0.12	0.2–5.7	1.1	1.6
			+	-	1118	0.01–0.5	0.06	0.12	0.16–5.7	1.3	1.7
			-	+	36239	0.01–0.5	0.23	0.25			
			-	-	489496	0.01–0.5	0.23	0.25			

^a ^A denotes a significant association, L denotes a significant linkage

^b ^Most heritable phenotypes (10%).

^c ^Phenotypes with >50% heritablility.

^d ^Relaxed thresholds: alpha levels do not account for testing of multiple phenotypes

We suspected that associations and linkages might be overrepresented for phenotypes that were more heritable. To examine this hypothesis, we rank ordered heritability estimates for the 3550 phenotypes and repeated the same classification procedure for both the top 10% most heritable phenotypes (rows 5 through 8 of Table 1) and all 108 phenotypes (rows 9 through 12 of Table 1) with heritability greater than 50%. We did not find any statistical differences in allele frequencies or β across bins under either subset of phenotypes.

As shown in Table 1, the number of SNPs that were both associated and linked is very low. This may reflect low power to detect associations due to the limited association sample size. To evaluate what type of distributions we may theoretically observe if more power was available, we relaxed the significance thresholds for linkage (LOD score > 3.3) and association (*p *< 4.74 × 10^-8^), reflecting a scenario in which only one phenotype was tested, as shown in the bottom four rows of Table 1. The number of SNPs tested under each linkage peak using this threshold was slightly lower than that using the most stringent threshold (mean = 2421, median = 2060). When relaxing the thresholds, we did not observe any significant differences among allele frequencies or β for associated SNPs within or outside of linkage regions.

Using our most conservative significance criteria we would expect 0.05 false-positive associations. With the more liberal criteria, we expect 178 (0.05 × 3,550) false-positive results. We observe 227 and 1200 associations, respectively, indicating that the majority of the observed associations (99.99% and 85.17%) are likely to be true associations. While choosing an even more liberal significance criteria could provide addition power to detect true associations, false-positive associations would become a greater proportion of the results. This would obscure any potential differences in allele frequencies and effect size as the false-positive results should reflect the underlying distribution of allele frequencies and effect sizes.

To investigate the statistical power available using this small sample of 57 independent subjects, we calculated power for a variety of allele frequencies and effect sizes and both significance criteria. Table 2 shows limited power for detecting effects with low allele frequencies (MAF = 0.05) and small to modest effects (β < 3.0) using the most stringent alpha level, 1.33 × 10^-11^. The more liberal threshold (α = 4.74 × 10^-8^) provides modest gains in power. In contrast, if expression data were available for all 210 unrelated individuals from HapMap, power of 0.8 or greater would exist for detecting more modest effects (>2.0) and also for larger effects with low allele frequencies.

**Table 2:** Estimate of power for genome-wide association across all phenotypes for 57 individuals

		α = 1.33 × 10^-11^			α = 4.74 × 10^-8^		
β	MAF	0.05	0.22	0.5	0.05	0.22	0.5
1.5		0	0	0.0006	0	0.0047	0.0261
2		0	0.0024	0.0333	0.0002	0.0641	0.298
2.5		0	0.0534	0.4646	0.0015	0.3791	0.888
3		0.0001	0.4415	0.9945	0.0075	0.8764	0.9999
3.5		0.0007	0.9694	0.9999	0.0305	0.9993	0.9999
4		0.0048	0.9999	0.9999	0.0999	0.9999	0.9999
4.5		0.0255	0.9999	0.9999	0.2586	0.9999	0.9999
5		0.1037	0.9999	0.9999	0.5175	0.9999	0.9999

## Discussion

Based on the CDCV hypothesis, we expected to see distinct differences in the distributions of allele frequencies and effect sizes between associated SNPs inside and outside regions of linkage. Our observation that allele frequencies did not differ between markers inside and outside linkage peaks was initially intriguing considering the CDCV hypothesis. However, because our data set included expression phenotypes of normal individuals, we should have an overrepresentation of common variants.

To confirm whether effect size played an important role for marker alleles inside versus outside linkage regions, we compared the regression coefficients β between the two distributions as an estimate of effect size. Although the use of β as a measurement of effect size might be questionable for linear regression, we minimized the variability in the ranges of values among all the traits through the use of log-transformed values. Our analysis showed that there were slight differences in the median effect size between the associated and linked versus associated-only bins, although these differences were not statistically significant.

An interesting phenomenon is the abundance of rare variants in the set of SNPs that were associated, which clearly deviates from the average MAF of 22% in the Illumina SNP panel, even though rare variants have low power (Table 2). A closer look at these data showed that single outliers were the driving force behind the regression models for these SNPs with a MAF of 0.01 or less. Because no other individuals shared the same genotype as the one with the phenotype outlier, it was difficult to determine whether the influence of the single data point was a biological or a spurious phenomenon in these situations, so such data were retained. However, one should be cautious when interpreting associated SNPs in Table 1 due to imprecision in the regression estimates (e.g., mean and median standard errors for β were 0.51 and 0.48 at the most stringent criterion) and the presence of outliers (i.e., the difference in the median and mean allele frequencies). A larger sample size would most likely resolve this question.

To accommodate the potential correlations among SNPs and phenotypes, we classified results using a more liberal threshold. Our power calculations, however, indicate that we had limited power to detect small to modest effects given our sample size. Extending our analysis to larger samples would require additional expression data and a large number of genotypes. For example, if similar expression data were available for all 210 unrelated HapMap subjects – subjects who already have extensive genotype data – we would have considerably greater power to detect more modest effects. Rare alleles (MAF < 0.05) with small effects (<1.5) would require more than 1500 subjects.

## Conclusion

In summary, there are at least two possible explanations for our observations. First, that the lack of difference between SNPs inside and outside linkage regions, is a real phenomenon. This would suggest that association effects are the same regardless of whether one is within or outside of a linkage region (i.e., with regard to allele frequencies and effect sizes). Another more compelling explanation is that with the GAW15 Problem 1 and publicly available data there was simply insufficient power to detect a difference between the distributions of either allele frequencies or effect sizes due to the small number of individuals available for association analyses.

## Competing interests

The author(s) declare that they have no competing interests.
